# What is the Prevalence of General Anxiety Disorder and Depression Symptoms in Semi-elite Australian Football Players: A Cross-Sectional Study

**DOI:** 10.1186/s40798-023-00587-3

**Published:** 2023-06-07

**Authors:** Anthony Henderson, Sarah Ann Harris, Troy Kirkham, Jonathon Charlesworth, Myles Calder Murphy

**Affiliations:** 1 Sportsmed Subiaco, St John of God Healthcare, Subiaco, WA Australia; 2 Institute for Health Research, The University of Notre Dame, Fremantle, WA Australia; 3 The West Australian Football Commission, Perth, WA Australia; 4 Sportsmed Glengarry, Duncraig, WA Australia; 5 Nutrition and Health Innovation Research Institute, School of Medical and Health Sciences, Edith Cowan University, Joondalup, WA Australia; 6 School of Health Sciences and Physiotherapy, The University of Notre Dame, Fremantle, WA Australia

**Keywords:** Mental health, Concussion, Epidemiology, AFL, Athlete, Wellbeing, Women’s sport

## Abstract

**Background:**

The prevalence of anxiety and depression symptoms in semi-elite Australian footballers is unknown. The primary objective of this study was to determine the prevalence of generalised anxiety disorder (GAD) and depressive symptoms in semi-elite Australian Football players. Our secondary objective was to explore the association between demographic and football-specific factors with GAD and depressive symptoms. A cross-sectional epidemiological study including 369 semi-elite Western Australian Football League (WAFL) players from the Men and Women’s 2022 season (*n* = 337 men, 91%) was conducted. Symptoms of depression were measured using the Patient Health Questionnaire-9 scale (PHQ-9) and symptoms of GAD with the GAD-7 scale.

**Results:**

Our response rate was 82.9%. Thirteen players had missing data. The prevalence of GAD symptoms was 8.5% in men and 28.6% in women (10% overall). The prevalence of depressive symptoms was 20% in men and 57% in women (23% overall). Being a woman (gender) was associated with a sevenfold increased risk of GAD and/or depression symptoms [odds ratio (OR): 7.33, 95% confidence interval (CI): 3.18–16.92; *p* < 0.001]. Players of ‘Aboriginal or Torres Strait Islander’ ethnicity were two times more likely to report GAD and/or depression symptoms in comparison to players of ‘Australian’ ethnicity (OR: 2.13; 95% CI: 1.01–4.49; *p* = 0.048). Concussion history was not a significant risk factor for GAD or depression symptoms.

**Conclusion:**

This study demonstrated that approximately 1 in 10 WAFL players met the diagnostic cut-off criteria for probable GAD, and 1 in 5 for probable depression. The prevalence for depression symptoms in this study were far higher than the national average in the comparative age bracket. WAFL women’s players also reported a substantially higher prevalence of GAD and depressive symptoms than men, and should be further investigated as a priority by the WAFL.

**Supplementary Information:**

The online version contains supplementary material available at 10.1186/s40798-023-00587-3.

## Key points

- The prevalence of anxiety and depression in semi-elite Australian footballers is unknown, as are those players most at risk.
- The prevalence of anxiety and depression symptoms in semi-elite Australian footballers appears higher than national averages.
- Women as well as players identifying as of ‘Aboriginal or Torres Strait Islander’ ethnicity are at the highest risk for anxiety and depression symptoms.

**Supplementary Information:**

The online version contains supplementary material available at 10.1186/s40798-023-00587-3.

## Introduction

Australian Football is a body collision sport that is played across Australia, with over 1.5 million participants [1]. Within one state jurisdiction (Western Australia), the semi-elite, state-level league includes the Western Australian Football League Women’s (WAFL-W) and Western Australian Football League Men’s (WAFL-M). The WAFL-W have seven league and five reserve teams, whereas the WAFL-M have ten league and nine reserves teams.

According to the Australian Bureau of Statistics (ABS) between 2020 and 2021, 21% (4.2 million) of Australians aged between 16 and 85 years had a mental disorder within the past 12 months [2]. Anxiety disorders were experienced by 16.8% of Australians aged between 16 and 85 years within the preceding 12 months and were more prevalent in females versus males (21.0% versus 12.4%) [2]. Specifically, 3.8% of the sample reported generalised anxiety disorder [2]. Affective disorders were experienced by 7.5% of Australians aged between 16 and 85 years within the preceding 12 months [2]. Specifically, 4.6% of the sample reported a depressive episode [2]. However, these figures are substantially higher amongst young people (aged 16–24 years) with almost twice the prevalence of mental disorders (39.6%) and with young females having a higher prevalence than young males (46.6% versus 31.2%) [2].

Semi-elite Australian football players are subject to many of the stressors typical of elite athletes [3], whilst also having to juggle work or study. However, research into the burden of mental health conditions in Australian athletes remains in its infancy. Kilic et al. (2021) reported that the prevalence of depressive symptoms in elite football (soccer) players was 7% and 8% for men and women, respectively [4], with similar rates seen in Japanese professional football players (9.4%) [5]. The 2020 Australian Football League Industry Mental Health and Wellbeing Report stated that the prevalence might even be as high as 17% in elite men [6]. However, there is a dearth of data on the prevalence of mental health conditions within the semi-elite Australian Football codes compared with international codes [7]. How generalisable these findings are to Australian athletes is unknown with clear differences in mental health burdens by country [8]. One study (*n* = 69) exploring risk factors for concussion within WAFL-M reported a prevalence of clinically relevant depressive symptoms of 8% [9]. Unfortunately, women are under-represented in sports medicine research [10], a problem also shared by health research into Aboriginal and Torres Strait Islander people [11]. This is a substantial problem with the rise of women’s sporting participation, particularly within Australian Football, considering that, when compared with men, women athletes report higher rates of mental health symptoms, including anxiety and depression [12, 13].

Within an athletic population, unique risk factors for mental health complaints can exist, when compared with the general population. An association between severe or long-term musculoskeletal injury and poor mental health, including depression and anxiety, has been reported in elite athletes, including athletes within the Australian Football League [14]. Recently an association between concussion and mental health outcomes in elite athletes has even been reported [15]. A 2018 systematic review of multiple sports indicated an association between concussion and depressive symptoms, with inconclusive evidence for the association with anxiety [16]. The relationship between other factors (e.g. socioeconomic status, sex, gender, ethnicity, age and family history) or Australian-Football-specific factors (e.g. being injured, relocation and history of concussion) is largely unreported.

Only by understanding the burden of mental health complaints, and the associated risk and protective factors, can measures be implemented to prevent and support players who experience mental health complaints.

### Objectives

The primary objective of this study was to determine the prevalence of general anxiety disorder (GAD) and depression symptoms in semi-elite Australian football players within the WAFL. The secondary objectives of our study were to explore the association between specific demographic (e.g. gender, age and pre-existing mental health illness) and football-specific factors (e.g. injury and concussion history) with self-reported GAD and depression symptoms.

## Methods

### Study Design

A cross-sectional epidemiological study was conducted between June 2022 and August 2022 during the WAFL (WAFL-W and WAFL-M) 2022 season.

### Setting

All WAFL-W (*n* = 7) and WAFL-M (*n* = 10) clubs were invited to participate within this study. One study author (AH) coordinated a single session for each WAFL-W and WAFL-M team that responded to our participation request (WAFL-W = 3 clubs, WAFL-M = 8 clubs) with the clubs’ respective football managers, where all players who attended for training were invited to complete an online survey.

### Participants

We included all WAFL-W and WAFL-M players who consented to participate within our study who were aged 18 years and over.

### Recruitment

A single author (AH) attended all participating WAFL-W and WAFL-M clubs to collect study data in a group session facilitated by the respective clubs football manager. Club visits were coordinated with the assistance of a study author (TK), who provided football club contacts and access. All players were provided a participant information sheet and given the opportunity to ask questions before providing consent using an online Qualtrics survey that was accessed by players on their smartphone via a QR code.

### Outcome Measures

All data was collected via an online Qualtrics survey, provided to participants in person at their football training and completed in the room, with assistance provided as needed by one study author (AH).

#### Demographic Data

We collected the following demographic data: age (years), gender (man/woman/non-binary/prefer not to say), ethnicity (based off ABS fields), currently injured (yes/no), history of concussion (yes/no), history of mental health diagnosis (yes/no).

#### Generalised Anxiety Disorder

The GAD-7 survey is a validated seven-item scale that is used in primary care and research to quantify the symptoms of GAD and has excellent internal consistency (Cronbach *α* = 0.92) and good test–retest reliability (intraclass correlation coefficient = 0.83) [17]. Each item is scored on a 0–3 scale with a total score out of 21 calculated [17]. A cut-off score of 10 and above is indicative of GAD (sensitivity = 89%, specificity = 82%) [17, 18] and was used as the cut-off for ‘GAD symptoms’ in this study.

#### Depression Symptoms

The Patient Health Questionnaire (PHQ-9) is a validated nine-item scale that is used in primary care and research to measure the symptoms of depression and has excellent internal consistency (Cronbach *α* = 0.89) and good test–retest reliability (intraclass correlation coefficient ≥ 0.80) [19]. Each item is scored using a 0–3 scale with a total score out of 27 calculated [19]. A cut-off score of 10 and over is indicative of a depressive disorder (sensitivity = 88%, specificity = 88%) [19, 20] and was used as the cut-off for ‘depression symptoms’ in this study.

### Sample Size Calculation

This study invited all WAFL-W and WAFL-M clubs in the 2022 season to participate. We estimated that each of the seven WAFL-W clubs had ~ 40 senior players and each of the 10 WAFL-M club’s had ~ 50 senior players, therefore, providing an estimated total available sample of 780 players, assuming each club chose to participate. Based on national ABS data with a precision of 0.05 and an expected prevalence of 12% for anxiety and 17% for depression (aged 15–34 years) [21] and adjusting for the finite population size [22], we required a minimum of 135 (GAD) to 170 (depression) participants to be included. Therefore, with our population sample of 780, and based on the scale with the greater number of participants required (i.e. depression) we would require a minimum of 170 players to be adequately powered.

### Statistical Analysis

All data were downloaded from Qualtrics, de-identified and inputted into a Microsoft Excel spreadsheet by a single study author (MM), and all statistical analysis was completed by a biostatistics research fellow (SH). Standard methods for examining descriptive data, including count (and percentage %) and means (standard deviation) were performed. Point prevalence was calculated.

Chi-square tests were used to explore the relationship between presence (yes/no) of GAD or depression symptoms and demographic variables (gender, ethnicity, previous mental health condition diagnosed, injury status and concussion history).

Three step-wise binary logistic regressions models were implemented to examine the association of self-reported concussion history (no/yes) and players with clinically relevant scores of (1) GAD, (2) depression and (3) GAD and/or depression (no/yes). Each model was adjusted for age (continuous), gender (woman, man or non-binary), mental health history (no/yes), current injury status (no/yes) and ethnicity (Australian, Aboriginal or Torres Strait Islander or other). PHQ-9 and GAD-7 scores (Model 1 and Model 2, respectively) were also adjusted for in the corresponding models. Data were analysed using SPSS for Windows (IBM SPSS, Version 27, Armonk, NY, USA). Statistical significance was identified at *p* < 0.05.

### Ethical considerations

To ensure that any players reporting significant mental health concerns were able to be followed up by the team medical doctor, within the consent section of the survey, players were asked whether they were happy for their results to be shared with their team doctor, and if so were able to provide their name and club. Thus, the data were required to be de-identified by a single study author.

## Results

### Response Rate

All 17 WAFL-W and WAFL-M teams were invited to participate in the study. Six (WAFL-W = 4, WAFL-M = 2) teams declined or did not respond to our request for participation. A total of 369 responses were received (WAFL-W = 32 players, WAFL-M = 337 players). Due to missing data in some survey items, 356 participants provided complete mental health survey responses (WAFL-W = 28 players, WAFL-M = 328 players). Therefore, for statistical purposes, all analyses are based off *n* = 356.

WAFL-W clubs each had approximately 15 players aged over 18 years in attendance for data collection (total possible sample across 3 clubs = 45 players) and WAFL-M each had approximately 50 players aged over 18 years in attendance for data collection (total possible sample across 8 clubs = 400 players). Therefore, our response rate for WAFL-W was 71% (32 of 45 players), and our response rate for WAFL-M was 84% (337 of 400 players), with an overall response rate of 83% (369 of 445 players).

### Demographic Data

Complete demographic data are presented within Table 1. The mean (SD) age of the sample was 22.1 (2.9) years [WAFL-M = 22 (2.9), WAFL-W = 22.2 (4.6) years]. Over four-fifths of the population identified as of ‘Australian’ ethnicity (86.2%), with 10.8% identifying as of ‘Aboriginal or Torres Strait Islander’ ethnicity. A total of 13% of participants had a previously diagnosed mental health condition (WAFL-M = 11.6%, WAFL-W = 31.3%). A total of 14% were currently injured at the time data collection, and almost half of participants (48.5%) self-reported a previous concussion.

**Table 1:** Participant demographics (*n* = 369)

		WAFL Men’s	WAFL Women’s	Total
Total	Sample	337 (91.3%)	32 (8.7%)	369 (100.0%)
Age (years)				
	M ± SD	22.05 ± 2.86	22.16 ± 4.64	22.06 ± 3.05
	Age range (years)	18–32	18–33	18–33
Ethnicity				
	Australian	292 (86.6%)	26 (81.3%)	318 (86.2%)
	Aboriginal Australian or Torres Strait Islander	35 (10.4%)	5 (15.6%)	40 (10.8%)
	Other	10 (3.0%)	1 (3.1%)	11 (3.0%)
Previously diagnosed with a mental health condition		39 (11.6%)	10 (31.3%)	49 (13.3%)
Currently injured		49 (14.5%)	3 (9.4%)	52 (14.1%)
Previously sustained a concussion		165 (49.0%)	14 (43.8%)	179 (48.5%)

Except for Total Sample, percent is based on each demographic variable per gender

*WAFL* Western Australian Football League, *M* mean, *SD* standard deviation

All data presented as *n* (%), unless stated otherwise

### Generalised Anxiety Disorder

The epidemiology of GAD symptoms are presented within Table 2 and detailed demographic breakdown within Table 3. Overall, 10.1% (*n* = 36) of players reported GAD symptoms, and proportionally, women (28.6%, *n* = 8) reported GAD symptoms more frequently than men (8.5%, *n* = 28). No differences in the GAD-7 score and concussion history (in both men and women) were observed (Table 4).

**Table 2:** Point prevalence of generalised anxiety disorder and depression symptoms in Western Australian Football League players

	Men	Women	Total
	(*n* = 328)	(*n* = 28)	(*n* = 356)
GAD and depression symptoms			
Point prevalence	26 (7.93%)	6 (21.43%)	32 (8.99%)
GAD-only symptoms			
Point prevalence	2 (0.61%)	2 (7.14%)	4 (1.12%)
Depression-only symptoms			
Point prevalence	39 (11.89%)	10 (35.71%)	49 (13.76%)

Point prevalence is presented as *n* (prevalence %)

**Table 3:** Differences in demographic data based on presence of generalised anxiety disorder or depression symptoms (*N* = 356)

		All participants (*n*)	GAD-7		PHQ-9	
			None	GAD symptoms	None	Depression symptoms
Total (*n*)		356	320	36	275	81
Age (M ± SD years)		22.08 ± 3.07	22.06 ± 2.93	22.19 ± 4.11	22.15 ± 3.07	21.84 ± 3.05
Gender	Men	328	300 (91.46%)	28 (8.54%)	263 (80.18%)	65 (19.82%)
	Women	28	20 (71.43%)	8 (28.57%)	12 (42.86%)	16 (57.14%)
Ethnicity	Australian	309	281 (90.94%)	28 (9.06%)	242 (78.32%)	67 (21.68%)
	Aboriginal or Torres Strait Islander	38	32 (84.21%)	6 (15.79%)	26 (68.42%)	12 (31.58%)
	Other	9	7 (77.78%)	2 (22.22%)	7 (77.78%)	2 (22.22%)
Previously diagnosed with mental health condition	No	310	284 (91.61%)	26 (8.39%)	250 (80.65%)	60 (19.35%)
	Yes	46	36 (78.26%)	10 (21.74%)	25 (54.35%)	21 (45.65%)
Currently injured and unable to complete standard football training	No	304	274 (90.13%)	30 (9.87%)	237 (77.96%)	67 (22.04%)
	Yes	52	46 (88.46%)	6 (11.54%)	38 (73.08%)	14 (26.92%)
Previously sustained a concussion	No	183	168 (91.80%)	15 (8.20%)	142 (77.60%)	41 (22.40%)
	Yes	173	152 (87.86%)	21 (12.14%)	133 (76.88%)	40 (23.12%)

All data presented as *n* (%), unless stated otherwise

Concussion history is self-reported by the athlete

GAD-7 general anxiety disorder possible score ranges from 0 to 21 (≥ 10 indicates probable GAD symptoms)

PHQ depressive symptoms possible score ranges from 0 to 27 (≥ 10 indicates probable depression symptoms)

*M* mean, *SD* standard deviation, *n* number, *GAD* generalised anxiety disorder, *GAD-7* Generalised Anxiety Disorder-Seven Items, *PHQ-9* Patient Health Questionnaire-Nine Items

**Table 4:** Differences in demographic, GAD and depressive symptom scores based on gender and concussion history (*N* = 356)

		Men		Women		Total	
	Concussion history	No	Yes	No	Yes	No	Yes
Total	Sample	165 (50.30%)	163 (49.70%)	18 (64.29%)	10 (35.71%)	183 (51.40%)	173 (48.60%)
Age (years)							
	M ± SD	21.78 ± 2.77	22.30 ± 2.96	22.17 ± 4.53	23.10 ± 5.59	21.82 ± 2.97	22.35 ± 3.15
Ethnicity							
	Australian	138 (83.64%)	148 (90.80%)	15 (83.33%)	8 (80.00%)	153 (83.61%)	156 (90.17%)
	Aboriginal Australian or Torres Strait Islander	21 (12.73%)	13 (7.98%)	2 (11.11%)	2 (20.00%)	23 (12.57%)	15 (8.67%)
	Other	6 (3.64%)	2 (1.23%)	1 (5.56%)	0 (0.00%)	7 (3.83%)	2 (1.16%)
Previously diagnosed with mental health condition							
	No	150 (90.91%)	140 (85.89%)	14 (77.78%)	6 (60.00%)	164 (89.62%)	146 (84.39%)
	Yes	15 (9.09%)	23 (14.11%)	4 (22.22%)	4 (40.00%)	19 (10.38%)	27 (15.61%)
Currently injured							
	No	140 (84.85%)	139 (85.28%)	15 (83.33%)	10 (100.00%)	155 (84.70%)	149 (86.13%)
	Yes	25 (15.15%)	24 (14.72%)	3 (16.67%)	0 (0.00%)	28 (15.30%)	24 (13.87%)
Mental health symptoms							
	No GAD or depression	132 (80.00%)	129 (79.14%)	7 (38.89%)	3 (30.00%)	139 (75.96%)	132 (76.30%)
	Probable GAD	1 (0.61%)	1 (0.61%)	2 (11.11%)	0 (0.00%)	3 (1.64%)	1 (0.58%)
	Probable depression	22 (13.33%)	17 (10.43%)	7 (38.89%)	3 (30.00%)	29 (15.85%)	20 (11.56%)
	GAD and depression	10 (6.06%)	16 (9.82%)	2 (11.11%)	4 (40.00%)	12 (6.56%)	20 (11.56%)
GAD-7 cut points (raw)							
	None	154 (93.33%)	146 (89.57%)	14 (77.78%)	6 (60.00%)	168 (91.80%)	152 (87.86%)
	Probable GAD	11 (6.67%)	17 (10.43%)	4 (22.22%)	4 (40.00%)	15 (8.20%)	21 (12.14%)
PHQ-9 cut points (raw)							
	None	133 (80.61%)	130 (79.75%)	9 (50.00%)	3 (30.00%)	142 (77.60%)	133 (76.88%)
	Probable depression	32 (19.39%)	33 (20.25%)	9 (50.00%)	7 (70.00%)	41 (22.40%)	40 (23.12%)
GAD-7 sum score							
	Mean ± SD	2.86 ± 3.60	3.16 ± 3.12	5.56 ± 3.33	6.60 ± 4.67	3.13 ± 3.66	3.36 ± 3.31
PHQ-9 sum score							
	Mean ± SD	2.82 ± 3.92	2.80 ± 2.96	4.39 ± 2.85	7.10 ± 4.28	2.98 ± 3.85	3.05 ± 3.20

*M* mean, *SD* standard deviation, *n* number, *GAD* generalised anxiety disorder, *GAD-7* Generalised Anxiety Disorder-Seven Items, *PHQ-9* Patient Health Questionnaire-Nine Items

All data presented as *n* (%), unless stated otherwise

Concussion history is self-reported by the athlete

GAD-7 general anxiety disorder possible score ranges from 0 to 21 (≥ 10 indicates probable GAD symptoms)

PHQ-9 depressive symptoms possible score ranges from 0 to 27 (≥ 10 indicates probable depression symptoms)

### Depressive Symptoms

The epidemiology of depressive symptoms is presented within Table 2 and detailed demographic breakdown within Table 3. Overall, 22.8% of players (*n* = 81) reported depressive symptoms, and proportionally, women (57.1%, *n* = 16) reported depression more frequently than men (19.8%, *n* = 65). No differences in the depression score and concussion history (in both men and women) were observed (Table 4).

### Variables Associated with Self-reported Mental Health Symptoms

Overall, the majority of players did not have GAD or depression symptoms per the self-report measures (76.1%, *n* = 271). Almost a tenth of all players (9.0%, *n* = 32) were classified with both depression and GAD per the self-report measure.

Chi-square tests demonstrated signification associations between GAD symptoms and gender (*χ*^2^ = 11.39, *p* ≤ 0.001) as well as a previous mental health diagnosis (*χ*^2^ = 7.86, *p* = 0.005). No associations were detected between GAD symptoms and ethnicity (*χ*^2^ = 1.73, *p* = 0.188), injury status (*χ*^2^ = 0.14, *p* = 0.712) or concussion history (*χ*^2^ = 1.52, *p* = 0.218).

Chi-square tests demonstrated signification associations between depression symptoms and gender (*χ*^2^ = 20.45, *p* ≤ 0.001) as well as a previous mental health diagnosis (*χ*^2^ = 15.76, *p* ≤ 0.001). No associations were detected between depressive symptoms and ethnicity (*χ*^2^ = 1.88, *p* = 0.170), injury status (*χ*^2^ = 0.60, *p* = 0.438) or concussion history (*χ*^2^ = 0.16, *p* = 0.692).

The results of binary logistic regression to determine the association of different variables with GAD and/or depressive symptoms are presented within Table 5. The final binary logistic regression model was statistically significant: *χ*^2^(5) = 27.74, *p* < 0.001. The model explained 11.2% (Nagelkerke R2) of the variance in GAD and/or depression symptoms and correctly classified 78.4% of cases. Of the four final predictor variables, two were statistically significant: gender and ethnicity (as shown in Table 5 and Additional file 1: Expanded Statistical Analysis).

**Table 5:** Predictors of GAD and depression symptoms in semi-elite Western Australian Football players (*n* = 356)

	B	SE	*p*-Value	Odds ratio (95%CI)
Constant	−0.60	0.95	0.526	0.549
Age (years)	−0.04	0.04	0.314	0.958 (0.88–1.04)
Gender (women)^a^	1.99	0.43	< 0.001*	7.334 (3.18–16.92)
Ethnicity^b^				
Aboriginal Australian or Torres Strait Islander	0.75	0.38	0.048*	2.126 (1.01–4.49)
Other	0.54	0.76	0.482	1.711 (0.38–7.66)
Concussion history^c^	0.16	0.27	0.546	1.174 (0.70–1.98)

^a^Compared with ‘Men’

^b^Compared with ‘Australian’

^c^Compared with ‘no concussion history’

^*^Significant at *p* < 0.05

Odds ratio derived from Exp(B) with 95% confidence interval. *SE* standard error; *GAD* generalised anxiety disorder

Model notes: Mental health history was excluded from the final model as this was accounted for by our primary outcome. Current injury status was excluded from the final model due to non-significance, and better model fit without the variable. Initial model details with mental health history and current injury status are included as Additional file 1

Identifying as a woman was associated with a greater than sevenfold increased risk of reporting GAD and/or depression symptoms in comparison to men (OR: 7.33, 95%CI: 3.18–16.92; *p* < 0.001). This finding did have relatively large confidence intervals, likely due to the smaller proportion of women; however, even the lower end of the confidence interval remained clinically significant. Players reporting an ‘Aboriginal or Torres Strait Islander’ ethnicity were two times more likely to report GAD and/or depression symptoms in comparison to players of ‘Australian’ ethnicity (OR: 2.13; 95%CI: 1.01–4.49; p = 0.048).

## Discussion

This is the first known study examining the epidemiology of self-reported depression and GAD symptoms among semi-elite Australian football players. Overall, our population reported a greater prevalence of depression (men and women) and GAD (women only) symptoms when compared with the general Australian population of a similar age [2]. As expected, players who reported a history of diagnosed mental health conditions or women (when compared with men) reported the greatest prevalence of GAD and/or depression symptoms. Furthermore, a greater proportion of players of ‘Aboriginal or Torres Strait Islander’ descent (16%) reported GAD compared with those players identifying as ‘Australian’ (9%). Similarly, 32% of players of ‘Aboriginal or Torres Strait Islander’ ethnicity reported depression symptoms, compared with 22% of players identifying as ‘Australian’ ethnicity. Concussion history was not associated with mental health prevalence or severity.

Women were associated with a sevenfold increased risk of reporting GAD and/or depression symptoms in comparison to men. Players of ‘Aboriginal or Torres Strait Islander’ ethnicity were twofold more likely to report GAD/depression symptoms in comparison to players reporting an ‘Australian’ ethnicity. Concussion history was not associated with reporting GAD/depression symptoms. Of specific concern was that nearly 1 out of every 2 (48.5%) players reported previously having a concussion in their history (which is higher than expected). Finally, 13.3% of our cohort had a previously diagnosed mental health condition, which is more prevalent than common musculoskeletal injuries, yet they likely receive far less management by club medical staff.

While there are numerous protective factors associated with engaging in physical activity and sport [23–25], many athletes may experience similar rates of depression to non-athletes. Sex differences are observed in the prevalence of depression at both the general population and athlete level [26], with females being more likely to report depressive symptoms than males. Our findings were consistent with existing research in other sports [4, 27], whereby the prevalence of depression in WAFL-W players (57.1%) was almost three times greater than in WAFL-M (19.8%) players, with one study of Australian elite soccer players and another in Icelandic athletes (football, handball and basketball) indicating women were almost twice as likely to experience moderate to severe depression symptoms than men [4, 27]. It is important to note that these findings need to be interpreted with caution due to the low number of women within our study.

The prevalence of depression symptoms was almost twice as high for men in our study (19.8%) when compared with ABS data of general Australian men with diagnosed affective disorders (16–24 years = 8.8%) [2] and Australasian male rugby athletes (12.6%) [28]. When compared with Kilic et al. 2021 (soccer), the prevalence of depression in our WAFL Men’s players was almost three times greater that elite Australian male soccer players [4] and over twice what was reported in Japanese professional football players [5]. However, our findings were similar to the prevalence rates observed in Australian elite athletes (23.6%) [3] and American male collegiate athletes (19.2%) [29], although these latter studies used the Centre of Epidemiologic Studies Depression Scale rather than PHQ-9. Furthermore, the screening tools (such as the PHQ-9) can overestimate true depression prevalence [30], which may explain the differences observed between the ABS data, which were collected via face-to-face interviews [2]. The lower rates of depression reported by the Dupreez et al. 2017 and Kilic et al. 2021 studies may also be due to different collection methods or sample sizes not being sufficient to quantify the true population prevalence [4, 28].

The prevalence of depression symptoms in our WAFL-W players was also substantially higher (57.1%) than Australian women with diagnosed affective disorders (16–24 years = 19.0%) [2], a previous study of Australian elite women’s soccer players (10.6%) [4] and Icelandic women athletes (12.2%) [27]. However, as with other areas of sports and exercise medicine, women remain substantially under-represented in research [10].

While men reported a higher level of depression symptoms than the general population, the WAFL-M players within this study reported a lower prevalence of GAD (9%) in comparison to Australian men with diagnosed anxiety disorders (16–24 years = 21.4%) [2]. However, the prevalence was greater than the reported rates of GAD in Australian elite men’s soccer players (4.7%) [4] and Japanese professional soccer players (3.1%) [5]. In contrast, women had a higher prevalence of GAD (29%) in comparison to the men within our study (9%), which is less than Australian women with diagnosed anxiety disorders (16–24 years = 41.3%) [2], and to the rates previously reported for elite Australian women’s soccer players (8.3%) [4]. It is unknown how these rates compare with other female Australian Footballers due to the paucity of research in this area.

Regular routine screening for these mental health conditions should be strongly encouraged [31], along with continued education regarding mental health and access to services, and the International Olympic Committee have an existing mental health tool for clinicians [32]. However, it must be acknowledged performing these assessments are time consuming, and semi-elite sporting organisations are unlikely to have the resourcing to implement this practice. Furthermore, the need for screening must be balanced with the burden placed upon athletes by regularly self-reporting data [33, 34]. These results clearly highlight that implementing interventions targeting management of mental health in all WAFL players should be seen as a priority, with a particular emphasis in WAFL-W and our Aboriginal and Torres Strait Islander’ athletes.

### Limitations

This study had a low number of WAFL-W players. The key reason for this low engagement was the number of women athletes playing in the senior league that were aged under 18 years (and therefore ineligible to participate). We had estimated at least 40 players able to participate per club; however, when we attended to collect data, there were only approximately 15 WAFL-W players in attendance aged over 18 years per club, which was substantially different to the WAFL-M. However, the investigation into women’s mental health has never been conducted in semi-elite Australia football, and our study still provides valuable data to stakeholders. Furthermore, with 369 participants, our study is one of the largest epidemiological studies for mental health for semi-elite athletes, and it is one of the few studies including both men and women athletes. Another limitation related to GAD and depressive symptom prevalence is our use of the GAD-7 and PHQ-9 to infer the prevalence of GAD and clinical depression. Whilst, this is commonly performed, and often the only feasible option for large, unfunded studies, these tools are not as accurate as a proper clinical examination.

A secondary limitation is the risk of self-report bias; however, this was mitigated due to the use of two validated mental health scales as well as dichotomous recall of concussion history (yes/no) rather than specific recollection of concussion frequency. However, the prevalence of self-reported concussion was higher than reported in previous epidemiological football research, and future studies should aim to clinically diagnose concussions using validated methods [35].

## Conclusion

This was the first study investigating the prevalence of symptoms of depression and GAD in semi-elite men’s and women’s Australian Football players. Approximately 1 in 10 players met the diagnostic cut-off criteria for GAD and 1 in 5 players for depression. Furthermore, women athletes were at a seven-times higher risk of mental health symptoms than men and players of 'Aboriginal or Torres Strait Islander' ethnicity were at a 2-times higher risk than players of ‘Australian’ ethnicity. These results clearly highlight that implementing interventions targeting management of mental health in all WAFL players should be seen as a priority, with a particular emphasis in WAFL-W and Aboriginal and Torres Strait Islander athletes.

## Supplementary Information

**Additional file 1**. Expanded Statistical Analysis﻿.

## Data Availability

The datasets generated and/or analysed during the current study are not publicly available due to the potential to identify individuals, given the limited number within the sample and that details such as age, height, weight or club affiliation are publicly available.

## Abbreviations

WAFL-W: Western Australian Football League Women’s
WAFL-M: Western Australian Football League Men’s
ABS: Australian Bureau of Statistics
GAD: General anxiety disorder
PHQ-9: Patient Health Questionnaire
SD: Standard deviation
