# Supplementary material for: What is the Prevalence of General Anxiety Disorder and Depression Symptoms in Semi-elite Australian Football Players: A Cross-Sectional Study
Source: Sports Med Open. 2023 Jun 7;9:42. doi: 10.1186/s40798-023-00587-3 (PMC10247656; doi:10.1186/s40798-023-00587-3)
Supplement: Supplementary file 1 — Additional file 1. Expanded Statistical Analysis﻿. [file 40798_2023_587_MOESM1_ESM.pdf]

TITLE: What is the prevalence of general anxiety disorder and depression symptoms in semi-elite Australian Football players: a cross-sectional study.

JOURNAL: Sports Medicine Open

Anthony Henderson[1]

Sarah Ann Harris[2]

Troy Kirkham[3]

Jonathon Charlesworth[4]

Myles Calder Murphy[1,5,6] *

[1]Sportsmed Subiaco, St John of God Healthcare, Subiaco, Western Australia, Australia

[2] Institute for Health Research, The University of Notre Dame, Fremantle, Western Australia, Australia

[3] The West Australian Football Commission, Western Australia, Australia.

[4] Sportsmed Glengarry, Duncraig, Western Australia, Australia

[5] Nutrition and Health Innovation Research Institute, School of Medical and Health Sciences, Edith Cowan University, Joondalup, Western Australia, Australia

[6] School of Health Sciences and Physiotherapy, The University of Notre Dame, Fremantle, Western Australia, Australia

**Model 1 - GAD and or Depression**				
	**B**	**SE**	**p-value**	**Odds Ratio (95%CI)**
Constant	*-0.637*	*0.963*	*0.508*	*0.529*
Concussion History	0.08	0.27	0.773	1.081 (0.64 - 1.84)
Sex (women)	1.89	0.44	<0.001*	6.629 (2.81 – 15.66)
Currently Injured	0.15	0.37	0.691	1.157 (0.56 – 2.38)
Mental Health History	1.16	0.35	0.001	3.200 (1.60 – 6.39)
Ethnicity				
Aboriginal or Torres Strait Islander	0.76	0.39	0.054	2.131 (0.99 – 4.60)
Other	0.59	0.80	0.463	1.799 (0.38 – 8.63)
Age (years)	-0.05	0.04	0.266	0.953 (0.87 – 1.04)

* Significant at p<0.05
SE: Standard Error; Odds Ratio: derived from Exp(B) with 95% confidence interval

**Model 2: GAD**				
	**B**	**SE**	**p-value**	**Odds Ratio (95%CI)**
Constant	*-6.001*	*1.685*	*<0.001*	*0.002*
Concussion History	0.76	0.45	0.089	2.146 (0.89 – 5.17)
Depression Score above the cut-point	3.82	0.58	<0.001*	45.586 (14.49 - 143.45)
Sex (women)	0.48	0.57	0.401	1.617 (0.53 - 4.96)
Currently Injured	-0.11	0.58	0.846	0.893 (0.28 - 2.80)
Mental Health History	0.31	0.51	0.548	1.358 (0.50 - 3.68)
Ethnicity				
Aboriginal or Torres Strait Islander	0.53	0.62	0.387	1.704 (0.51 - 5.70)
Other	2.15	1.13	0.058	8.548 (0.93 - 78.26)
Age (years)	0.05	0.07	0.478	1.050 (0.92 - 1.20)

* Significant at p<0.05
SE: Standard Error; Odds Ratio: derived from Exp(B) with 95% confidence interval

**Model 2b: Final model for GAD outcome**				
	**B**	**SE**	**p-value**	**Odds Ratio (95%CI)**
Constant	*-5.95*	*1.67*	*<0.001*	*0.003*
Concussion History	0.77	0.45	0.084	2.167 (0.90 - 5.21)
Depression Score above the cut-point	3.84	0.58	<0.001*	46.676 (14.97 – 145.53)
Sex (women)	0.54	0.56	0.340	1.708 (0.57 – 5.13)
Ethnicity				
Aboriginal or Torres Strait Islander	0.54	0.61	0.377	1.718 (0.52 – 5.72)
Other	2.10	1.14	0.065	8.145 (0.88 – 75.45)
Age (years)	0.05	0.07	0.486	1.049 (0.92 – 1.20)

* Significant at p<0.05
SE: Standard Error; Odds Ratio: derived from Exp(B) with 95% confidence interval

**Model 3: Depressive outcome**				
	**B**	**SE**	**p-value**	**Odds Ratio (95%CI)**
Constant	*-0.344*	*1.190*	*0.772*	*0.709*
Concussion History	-0.12	0.32	0.703	0.886 (0.47 – 1.65)
GAD Score above the cut-point	3.78	0.58	<0.001*	44.014 (14.10 - 137.43)
Sex (women)	1.40	0.49	0.004*	4.070 (1.56 - 10.58)
Currently Injured	0.15	0.41	0.712	1.165 (0.52 - 2.62)
Mental Health History	1.09	0.41	0.008*	2.974 (1.32 - 6.69)
Ethnicity				
Aboriginal or Torres Strait Islander	0.34	0.47	0.468	1.404 (0.56 - 3.52)
Other	-1.11	1.42	0.435	0.329 (0.02 - 5.35)
Age (years)	-0.08	0.05	0.166	0.927 (0.83 - 1.03)

* Significant at p<0.05

SE: Standard Error; Odds Ratio: derived from Exp(B) with 95% confidence interval

**Model 3b: Final model for depressive outcome**				
	**B**	**SE**	**p-value**	**Odds Ratio (95%CI)**
Constant	*-0.46*	*1.15*	*0.690*	*0.631*
Concussion History	-0.05	0.31	0.866	0.949 (0.51 - 1.75)
GAD Score above the cut-point	3.78	0.57	<0.001*	43.995 (14.37 - 134.67)
Sex (women)	1.49	0.48	0.002*	4.455 (1.75 - 11.31)
Ethnicity				
Aboriginal or Torres Strait Islander	0.38	0.46	0.406	1.469 (0.59 - 3.64)
Other	-1.10	1.33	0.406	0.332 (0.02 - 4.47)
Age (years)	-0.06	0.05	0.229	0.938 (0.85 - 1.04)

* Significant at p<0.05

SE: Standard Error; Odds Ratio: derived from Exp(B) with 95% confidence interval
